# Interventional closure of a bronchopleural fistula in a 2 year old child with detachable coils

**DOI:** 10.1186/s12887-022-03298-y

**Published:** 2022-05-05

**Authors:** Winfried Baden, Michael Hofbeck, Steven W. Warmann, Juergen F. Schaefer, Ludger Sieverding

**Affiliations:** 1 Department Paediatrics 2, Pulmonology, Cardiology, Intensive Care, Children’s Hospital, University of Tuebingen, Hoppe-Seyler-Strasse 1, 72076 Tuebingen, Germany; 2 Department Paediatric Surgery and Paediatric Urology, Children’s Hospital, University of Tuebingen, Tuebingen, Germany; 3 Department Radiology, Division of Paediatric Radiology, University Hospital, Tuebingen, Germany

**Keywords:** Pneumonia, Pleural empyema, Bronchopleural fistula, Coil occlusion, Interventional bronchoscopy

## Abstract

**Background:**

Bronchopleural fistula (BPF) is a severe complication following pneumonia or pulmonary surgery, resulting in persistent air leakage (PAL) and pneumothorax. Surgical options include resection, coverage of the fistula by video-assisted thoracoscopic surgery (VATS), or pleurodesis. Interventional bronchoscopy is preferred in complex cases and involves the use of sclerosants, sealants and occlusive valve devices.

**Case presentation:**

A 2.5-year-old girl was admitted to our hospital with persistent fever, cough and dyspnoea. Clinical and radiological examination revealed right-sided pneumonia and pleural effusion. The child was started on antibiotics, and the effusion was drained by pleural drainage. Following removal of the chest tube, the child developed tension pneumothorax. Despite insertion of a new drain, the air leak persisted. Thoracoscopic debridement with placement of another new drain was performed after 4 weeks, without abolishment of the air leak. Bronchoscopy with bronchography revealed a BPF in right lung segment 3 (right upper-lobe anterior bronchus). We opted for an interventional approach that was performed under general anaesthesia during repeat bronchoscopy. Following bronchographic visualisation of the fistula, a 2.7 French microcatheter was placed in right lung segment 3 (upper lobe), allowing occlusion of the fistula by successive implantation of 4 detachable high-density packing volume coils, which were placed into the fistula. Subsequent bronchography revealed no evidence of residual leakage, and the chest tube was removed 2 days later. The chest X-ray findings normalized, and follow-up over 4 years was uneventful.

**Conclusions:**

Bronchoscopic superselective occlusion of BPF using detachable high-density packing large-volume coils was a successful minimally invasive therapeutic intervention performed with minimal trauma in this child and has not been reported thus far. In our small patient, the short interventional time, localized intervention and minimal damage in the lung seemed superior to the corresponding outcomes of surgical lobectomy or pleurodesis in a young growing lung, enabling normal development of the surrounding tissue. Follow-up over 4 years did not show any side effects and was uneventful, with normal lung-function test results to date.

**Supplementary Information:**

The online version contains supplementary material available at 10.1186/s12887-022-03298-y.

## Background

Bronchopleural fistulae (BPFs) are rare but severe complications of pneumonia, lung abscess or pleural empyema. They may also occur following thoracic surgical procedures resulting in persistent air leakage (PAL). Surgical options include resection, video-assisted thoracoscopic surgery (VATS), coverage of the fistula, or pleurodesis. Interventional bronchoscopy is preferred in complex cases. Endoscopic interventional treatment options in adults comprise a variety of procedures, including implantation of devices such as volume-reduction valves [1] or ASD occluders [2], following fistula visualization and balloon sizing under fluoroscopy. The devices must achieve immediate, airtight occlusion and must subsequently be removed. Risks comprise valve malpositioning and device expectoration during cough [3]. Furthermore, sclerosants are used to achieve pleurodesis by using talc [4], ethanol [5], silver nitrate [6], polyethylene glycol [7], tetracycline, doxycycline [8], minocycline [4] or bleomycin [9]. They have to be administered strictly locally, as they provoke severe inflammatory reactions, which might cause severe damage to other pulmonary segments. Complications include chest pain, fever, acute lung injury, and/or subsequent interstitial lung disease [9]. In cases of persistent pneumothorax, the altered lung may be unable to be re-expanded [10]. Autologous blood instillation into the pleural cavity for coverage of the fistula is of limited success, resulting in a pleural callosity and may be complicated by chest tube clots, pleuritis and empyema [10, 11]. While this treatment is easy to perform, success is uncertain, requiring prolonged periods of chest tube drainage. Sealants such as cyanoacrylate [12, 13], fibrin glue [14–17], albumin-glutaraldehyde glue [18], hydrogel [19] or oxidized cellulose [20] have also been used for fistula occlusion. Since interventional occlusion with detachable metallic coils usually does not achieve immediate airtight occlusion, it has been recommended to combine the use of these coils with additional topical sealants [21]. While this technique is associated with the risk of displacement of the sealant, long-term animal studies have shown complete resorption of the glue within 3 months [17]. To date, interventional procedures for occlusion of BPFs have been performed exclusively in the adult population.

## Case presentation

**Fig. 1:**
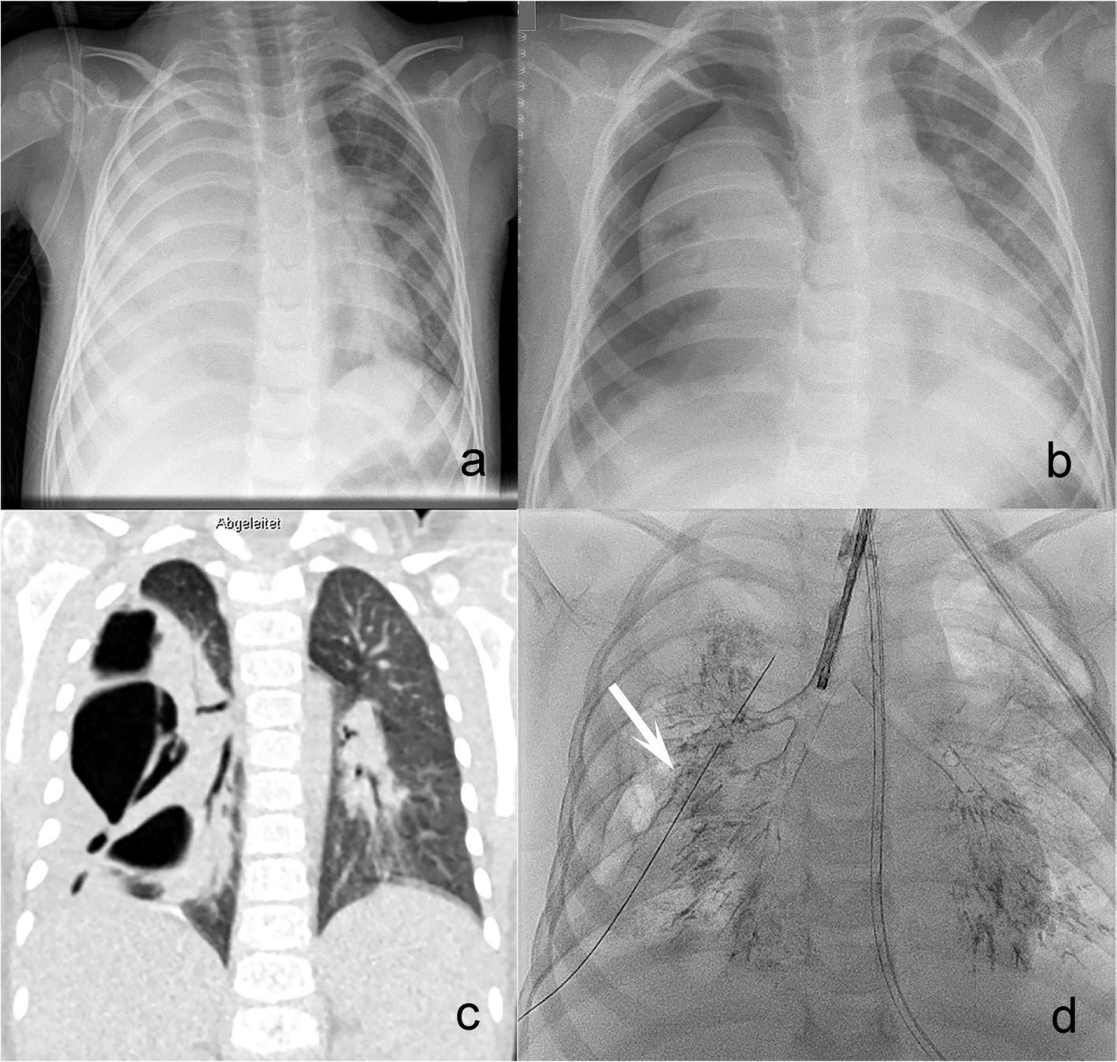
**a** Chest Xray with right sided pneumonia and effusion. **b** Chest Xray with tension pneumothorax right sided. **c** CT-Scan, coronary slice, pneumonia with dystelectasis, lateral pneumothorax and right sided fistula. **d** Bronchography with proof of PAL bronchus 3r (white arrow)

We report on a 2 ½-year-old female child who presented in a reduced general condition with a fever of 40 °C, tachypnoea, marked respiratory distress and cough. Clinically and radiologically, right-sided pneumonia and pleural effusion were diagnosed (Fig. 1a). The child was transferred to the PICU, started on antibiotics, ventilated noninvasively and treated by chest tube insertion. Two days later, immediately after removal of the drainage, the patient developed tension pneumothorax (Fig. 1b). A new drain was placed, but the air leak persisted for the next 3 weeks, and weaning from drainage was impossible. A CT scan (Fig. 1c) displayed a partially collapsed right lung with dystelectasis and septate pneumothorax. As surgical thoracoscopic debridement with insertion of a new drain remained unsuccessful, we performed a diagnostic bronchoscopy 3 days later with bronchographic confirmation of a BPF in right-sided segment 3 (Fig. 1d, Additional video file 1). The girl was monitored for spontaneous breathing in the ICU.

**Fig. 2:**
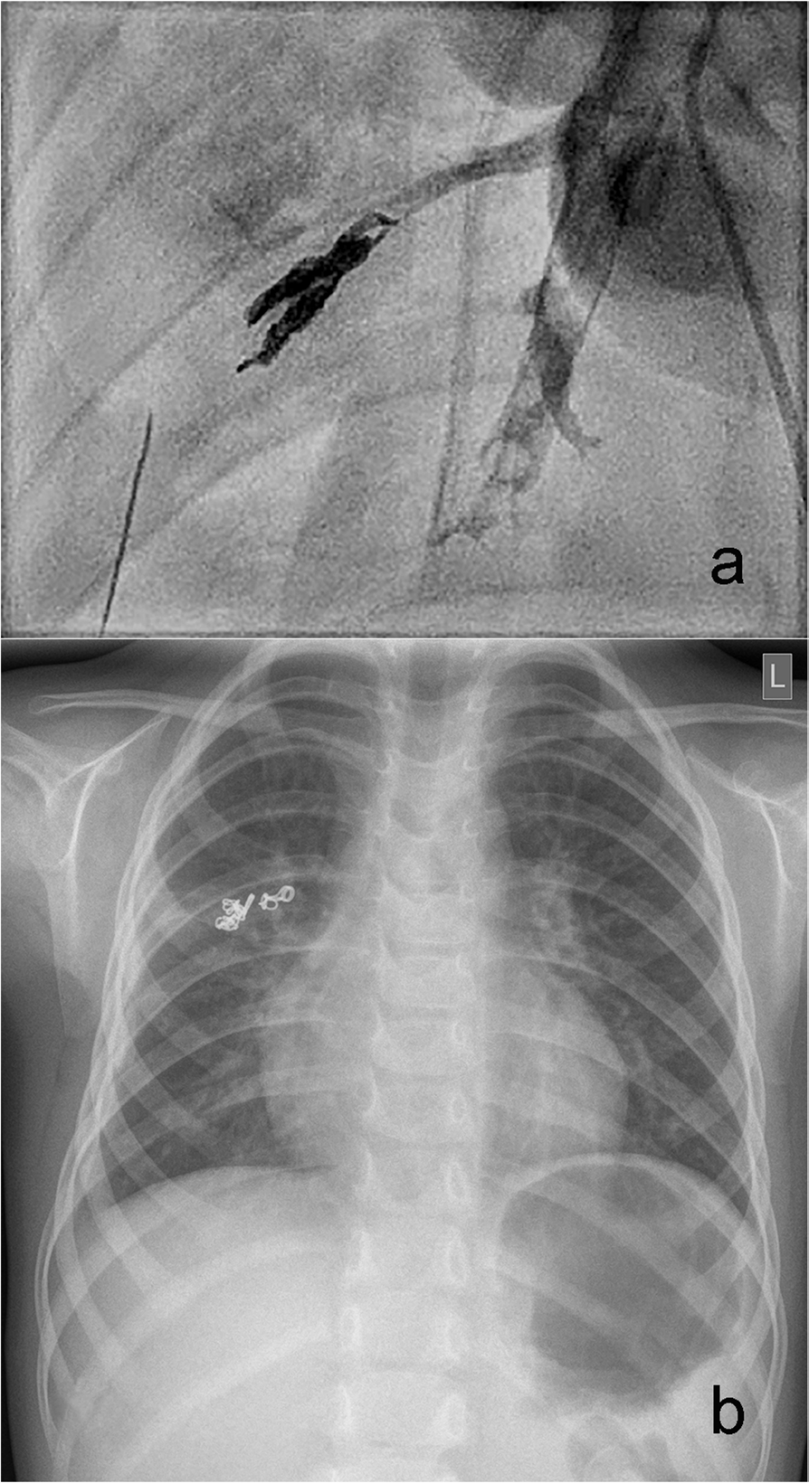
**a** Coiling Bronchography after coiling PAL in bronchus 3r. **b** Chest Xray control 3 months after endobronchial intervention

Intervention: After interdisciplinary consultation, we opted for interventional occlusion of the fistula, applying a vascular occlusion technique. In contrast to endovascular occlusion procedures, a vascular plug requiring thrombus formation inside the device could not be used to achieve complete occlusion. We therefore decided to use detachable large-volume coils, which can be applied via microcatheters (Ruby Coil Penumbra®, Alameda California USA) for the occlusion of cardiovascular malformations [22, 23]. Since these coils allow a high packing density, they offer a high probability of primary airtight occlusion and complete abolishment of air leakage. The bronchoscopic intervention was performed under general anaesthesia starting with bronchographic visualisation of the BPF (contrast media (Ultravist® 400)). A 4 French catheter (Glidecath Terumo®, Tokyo Japan) was advanced into segment 3 as a guiding catheter to allow selective placement of a 2.7 French microcatheter (Progreat Terumo®, Tokyo Japan). Superselective embolization of the fistula was achieved by successive placement and dense packing of 4 large-volume detachable coils (2 mmx1 cm, 3 mmx5 cm and 4 mmx6 cm Ruby soft coils and 3 mmx5 cm Ruby Standard Coil®, Ruby Coil Penumbra®, Alameda California USA). Repeat bronchography revealed complete occlusion of the fistula without evidence of residual leakage (Fig. 2a). Two days later, the drain was removed, and the child was extubated. During follow-up over 4 years, the coil position remained unchanged, the X-ray findings normalized, and the child was clinically asymptomatic (Fig. 2b) with normal lung function both in spirometry and bodyplethysmography. She shows normal total lung capacity and vital capacity, normal oxygen saturation and normal exercise tolerance in daily life. Three supplementary movie files show these diagnostics and procedure in more detail (see Additional files 1, 2 and 3).

## Discussion and conclusions

Air leaks due to BPF are associated with high morbidity (up to 57% for resectional surgery or pneumonia) and significant mortality (16–72%) [1, 24–28]. Such leaks persisting longer than 5–7 days [1] are defined as ‘persistent air leaks (PALs)’, specified as the entry of air into the pleural space originating either from central airways (BPF) [1] or from the periphery (alveolo-pleural fistula). For diagnostic confirmation, sequential bronchial blocking or methylene blue staining in the pleura are recommended [29]. In our case, we achieved exact defect localization by endoscopically controlled selective bronchography.

Although a variety of therapeutic options have been published, they have to be adjusted to the origin and size of the fistula. Fistulae developing after surgical resection therapy due to cancer or malformation may be easier to occlude surgically than fistulae developing as a complication of severe pneumonia or empyema. In addition, a fistula of > 8 mm should be primarily closed surgically [28], although endoscopic intervention may be less invasive than a thoracotomy. According to a review of the literature and to the recommendations and guidelines of the American Thoracic Society (ATS, 2001) [30], British Thoracic Society (BTS, 2010) [27] and European Association for Cardio-Thoracic Surgery (EACTS, 2015) [31], the treatment of first choice is a conservative approach with drainage by a chest tube (suction or water seal) and observation [1]. A digital measurement of air leak flow by means of newer electronic chest drainage systems is helpful [1]. In cases of failure of this conservative approach, surgical therapy is recommended. Surgical treatment (open or as VATS) comprises either resection of any bullae, blebs or visible anomalies; coverage of the fistula [32, 33]; or mechanical or chemical pleurodesis [1]. However, in patients with BPF following surgical procedures, endoscopic therapy has been recommended as the treatment of first choice since it has proven superior to resurgery [25]. As discussed above, endoscopic interventional treatment options in adults comprise a variety of procedures, including occluding device implantation and topical administration of sclerosants or sealants. Plugs and valves are large in size and derived from adult medicine and COPD therapy; however, Amplatzer® devices and coils used in paediatric cardiology may be used in older children. Valves have to be removed after healing, can be expectorated by accident and are too large for small children. In our patient, we did not favour the use of topical sclerosant administration due to the risk of accidental malplacement of the sealant in the presence of rather small airways.

With respect to the age and small size of the bronchial anatomy in our patient, we decided on a hitherto unpublished interventional approach of bronchoscopic fistula occlusion by implantation of detachable high-density packing, high-volume coils (Penumbra®). They are relatively small, become progressively softer from their distal to proximal end rather than being of uniform stiffness, exhibit a tight conformational structure and have a more robust stretch-resistance platform. Normally indicated for aneurysms and arterial and venous embolization in the peripheral vasculature, they have proven to be safe for endovascular procedures [22, 34].

These coils can be delivered via microcatheters allowing superselective occlusion of small structures and have been used in paediatric cardiology and neuroradiology. Due to the dense 3D packing of 4 consecutive coils, we were able to achieve immediate airtight occlusion with minimal trauma to the pulmonary tissue, avoiding the requirement of the additional application of fibrin glue or other sealants. Rapid removal of the drain and discharge from the hospital was possible, with uneventful follow-up over a 4-year period. As only 2 subsegments of segment 3 of the right upper lobe had been occluded, and with respect to the Euler-Liljestrand reflex, no VQ mismatch was expected; moreover, callous scarring enabled compensatory enlargement of the resting neighbouring lobe segments.

In summary, we report bronchoscopic superselective occlusion of a BPF using detachable high-density packing large-volume coils as a successful minimally invasive therapeutic intervention in a 2.5-year-old girl. According to our experience, detachable high-density packing microcoils, which can be placed selectively via microcatheters, may represent an attractive alternative for interventional occlusion of BPFs in small children. This minimally invasive therapeutic intervention has advantages compared to surgery due to its minimally invasive nature associated with only minor trauma to the lung tissue. Since the method described here can be performed in adults under local anaesthesia, it could be a therapeutic option for this life-threatening complication in selected patients who are at high risk for surgery under general anaesthesia. Further experience will be necessary to recommend this new method for standard care in the future.

## Supplementary Information

**Additional file 1: Additional movie file 1.** Bronchography showing BPF in right sided segment 3

**Additional file 2: Additional movie file 2.** Procedure of coiling the BPF

**Additional file 3: Additional movie file 3.** Control bronchography after interventional coiling

## Data Availability

Movie files are available as Additional files 1, 2 and 3. Patient records, CTs and CRs are with the corresponding author.

## Abbreviations

ATS: American Thoracic Society
BPF: Bronchopleural fistula
BTS: British Thoracic Society
CT: Computed tomography
CR: Chest X-ray
EACTS: European Association of Cardio-Thoracic Surgery
PAL: Persistent air leak
VATS: Video-assisted thoracoscopic surgery
